# *Echinococcus granulosus* hydatid cyst location is modified by *Fasciola hepatica* infection in cattle

**DOI:** 10.1186/s13071-018-3128-6

**Published:** 2018-10-10

**Authors:** Caroll Stoore, Constanza Andrade, Christian Hidalgo, Felipe Corrêa, Mauricio Jiménez, Marcela Hernandez, Rodolfo Paredes

**Affiliations:** 1 Escuela de Medicina Veterinaria, Facultad de Ciencias de la Vida, Universidad Andres Bello, Santiago, Chile; 2 Laboratorio de Biología Periodontal, Facultad de Odontología, Universidad de Chile, Santiago, Chile; 3 Facultad de Ciencias de la Salud, Universidad Autonoma de Chile, Santiago, Chile

**Keywords:** *Echinococcus granulosus*, *Fasciola hepatica*, Polyparasitism, Hydatid cyst localization

## Abstract

**Background:**

Natural parasite infection occurs in wild and domestics animals with more than one parasite species at the same time, generating an infection called polyparasitism. Cystic echinococcosis reports are usually based only on infection with *Echinoccocus granulosus* leaving aside other internal parasitoses that could modulate both the immune response and pathogenesis of the natural infection. *Fasciola hepatica* is another cosmopolitan parasite in ruminants with a similar distribution to *E. granulosus* in different parts of the world, but no information of the effect of co-infection with *E. granulosus* has been described. The aims of this report were to establish *E. granulosus* prevalence and explore the association of *F. hepatica* co-infection and natural *E. granulosus* infections in cattle.

**Results:**

From 1725 animals, the prevalence of *E. granulosus* and *F. hepatica* was 21.16 and 51.3%, respectively. Considering both infections, older cattle (> 4 years) presented higher prevalence compared to younger animals. In *E. granulosus*-infected cattle, 5.21% had fertile cysts, 71.78% infertile cysts, and in 23.01% cysts were smaller than 1 cm in diameter. Considering cyst location, 39.72% had lungs cysts, 24.72% had liver cysts and 36.94% had cysts in both organs. Cyst location significantly differed between age groups: 44.68% of younger animals had cysts only in the lungs, while older animals presented hydatid cyst in the lungs and liver simultaneously (44.15%). With *E. granulosus* infection alone, 30.26% of cysts were found in the lungs, 31.79% in the liver and 37.95% in both organs. Regarding the co-infection of *E. granulosus* with *F. hepatica*, the proportion was significantly different (*P* < 0.05) with most animals having cysts only in the lungs (49.41%) and a lower level of liver infection (15.88%). Analyzing organ cyst distribution and *F. hepatica* absence/presence ratio within each cyst type, small cysts showed the highest difference in ratio.

**Conclusions:**

To the best of our knowledge, this is the first report indicating that *F. hepatica* co-infection in cattle could be affecting the instate of hydatid cysts in the liver, displacing toward lung localization, suggesting an antagonistic relationship.

## Background

Co-infection with different parasite species in the same host (also known as polyparasitism) is a well-documented fact in medical, veterinary and zoological literature. Most of the animals that live in the wild and humans of rural areas can be hosts of many concurrent parasite species [1]. However, the synergistic or antagonic relationship that different parasite species can have within the same host remains poorly studied [2]. Helminth parasites are a very diverse group of animals that are classified in four taxonomic groups: nematodes, trematodes, cestodes and acanthocephalans [3]. In cattle, two parasites usually represent a frequent infection: the cestode *E. granulosus* (*sensu lato*) and the trematode *F. hepatica*. *Echinococcus granulosus* (*s.l.*) has an indirect life-cycle, with ruminants as intermediate hosts, dogs and other canids as definitive hosts, and humans as dead-end hosts [4]. The metacestode stage called hydatid cysts develops in the viscera (mainly lungs and liver) of the intermediate hosts [5], causing a disease known as cystic echinococcosis. *Fasciola hepatica* also has an indirect life-cycle; however, herbivores act as the definitive hosts, with the adult worms located in the bile ducts [6].

*Echinococcus granulosus* (*s.l*.) is composed of *E. granulosus* (*sensu stricto*) (genotypes G1-3), *E. equinus* (genotype G4), *E. ortleppi* (genotype G5), *E. canadensis* (genotypes G6-8/G10) and *E. felidis* (“lion strain”), with *E. granulosus* (*s.s.*) being the most commonly distributed worldwide [7].

Although *E. granulosus* (*s.l*.) is able to infect a wide range of mammalian hosts, the metacestode stage has a different capacity to produce protoscoleces, the stage infective to the definitive host. For unknown reasons, the parasite can, in some animals, produce protoscoleces inside the cyst, generating a fertile hydatid cyst, but other animals with cystic echonococcosis possess cysts without protoscoleces called infertile hydatid cysts [8, 9]. Since cyst fertility is associated with the size of the hydatid cyst [4], there is a subset of hydatid cysts that are too small to be classified as either fertile or infertile. The cellular and molecular mechanisms involved in the process of cyst fertility remain unknown [10]. In cattle, hydatid cyst fertility status ranges from 0 to 96% in different parts of the world [11–22] and infection with *F. hepatica* is common in many parts of the world. However, there are no studies on relationships between *E. granulosus* (*s.l.*) and *F. hepatica* in co-infections.

Although belonging to different higher-level flatworm taxa, both parasites exhibit common traits regarding their interaction with the mammalian host; as such, serum of animals infected experimentally with *E. granulosus* (*s.l.*) can recognize *F. gigantica* antigens in immunoassays [23] but there are no specific data on cross-reaction with *F. hepatica*. However, both parasites have the ability to uptake host glycolipids [6] which could explain the latter. Reports of polyparasitism involving *E. granulosus* (*s.l.*) are scarce. There is only one report that includes the interaction with *Schistosoma mansoni* (a trematode), where in murine models simultaneous concomitant infection lead to higher IFN-γ profiles, displaying a T_H_1 response; however, adding *E. granulosus* infection seven weeks after *S. mansoni* infection led to significant lower IL-10 production, changing the immune profile to a T_H_2 response [24]. Here, we provide the first report that in bovines infected with *E. granulosus* (*s.l.*), the presence of co-infection with *F. hepatica* is associated with changes in the hydatid cysts localization.

## Methods

### Sampling design

A total of 1725 cattle were examined for the presence of hydatid cyst and *F. hepatica* infection at *post-mortem* inspection in a Region Metropolitana slaughterhouse, Chile. In routine slaughtering, animals were individually identified, age and sex was recorded, and visceral organs of each animal, mainly the lungs and liver, were visually examined, palpated and incised along with official veterinarian inspectors for the presence of hydatid cysts and *F. hepatica*.

*Fasciola hepatica* diagnosis was made either by direct visualization of adult parasites in bile ducts, or by *F. hepatica* compatible lesions such as enlarged and thickened bile ducts, calcification of bile ducts, black parasitic material and black lymph nodes in the liver: signs of chronic *F. hepatica* infection.

Suspected cystic samples were removed from the infected organ, placed in separate polythene bags and transported in an isothermal container within 3 h to Universidad Andres Bello Veterinary School for further examination. For hydatid cysts confirmation and fertility determination, cysts were microscopically examined as previously described [25]. *Echinococcus granulosus* (*s.l.*) genotyping in hydatid cyst samples was determined as previously reported [26]. Briefly, DNA was extracted from fertile, infertile and small hydatid cysts from both livers and lungs. The *cox*1 mtDNA was amplified and sequenced, and a 345-nucleotide consensus sequence was used for comparison analysis.

### Study groups

Animals were classified according to their age, hydatid cyst type and location. By age range, individuals were divided into two groups: 4 years-old or younger (≤ 4 years) and over 4 years of age (> 4 years). Cysts were classified into 3 types: small cysts (< 1 cm in diameter); fertile cysts (with protoscoleces); and infertile cysts (> 1 cm in diameter and without protoscoleces). Animals were also separated into 3 groups according to the location of the hydatid cysts: in lungs only; in liver only; and in both organs simultaneously. All groups were also separated by their *F. hepatica* co-infection status.

### Data analysis

Data were recorded in an Excel 2010 datasheet and analyzed with RStudio IDE version 1.0.136 and R version 3.3.3 for statistical associations among variables using a Chi-square test. Logistic regressions were performed using STATA v.12 software (StataCorp, College Station, TX, USA). Statistical significance was considered when *P*-values were below the 0.05 threshold.

## Results

### Prevalence of *E. granulosus* and *F. hepatica*

**Fig. 1:**
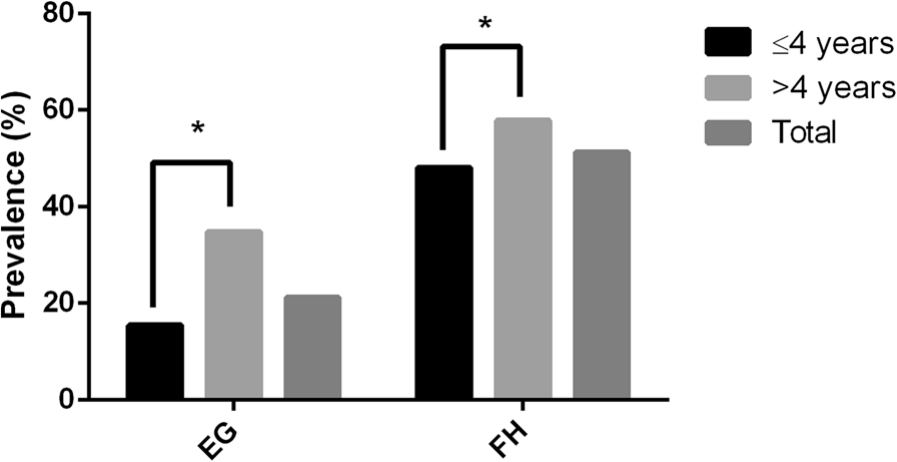
*Echinococcus granulosus* (EG) and *Fasciola hepatica* (FH) overall prevalence per age group in inspected cattle **(***n* = 1725)**.** Data shown as percentage of infected/ total examined cattle in the respective categories. *P < 0.05 (Chi-square test)

Of the animals examined, 1217 were 4 years of age or under, and 508 were over 4 years-old. The overall prevalence of *E. granulosus* and *F. hepatica* was 21.16% (95% CI: 19.25–23.16%) and 51.3% (95% CI: 48.92–53.69%), respectively. For both parasites, older cattle (> 4 years-old) had a significantly higher prevalence than younger animals (≤ 4 years-old) (*E. granulosus*: *χ*^2^ = 80.81, *df* = 1, *P* < 0.0001; *F. hepatica*: *χ*^2^ = 12.56, *df* = 1, *P* = 0.0002) (Fig. 1). Separating co-infected animals, which represented 9.86% (*n* = 170) of the sampled population, *E. granulosus*-only infected cattle (*n* = 195, 11.3%) remains higher in older animals (*χ*^2^ = 22.72, *df* = 1, *P* < 0.0001), as in co-infected cattle (*χ*^2^ = 52.63, *df* = 1, *P* < 0.0001), but not in animals with *F. hepatica*-only infection (*n* = 715, 41.45%) where no significant difference between age groups was found (*χ*^2^ = 0.4273, *df* = 1, *P* = 0.5133) (Table 1).

**Table 1:** *Echinococcus granulosus*, *Fasciola hepatica* and co-infection prevalence, per age range classification of slaughtered cattle

	Examined	EG only			FH only			EG and FH co-infected		
	*N*	*n*	%	95% CI	*n*	%	95% CI	*n*	%	95% CI
Overall	1725	195	11.30	9.90–12.89	715	41.45	39.15–43.79	170	9.86	8.54–11.35
≤ 4 years	1217	109	8.96	7.48–10.69	507	41.66	38.92–44.45	79	6.49	5.24–8.02
> 4 years	508	86	16.93	13.92–20.44	203	39.96	35.79–44.28	91	17.91	14.82–21.48

*Abbreviations*: *FH Fasciola hepatica*, *EG Echinococcus granulosus*, *N/n* number of animals by category, %, percentage of infection(s) positive animals of the total of animals examined by category

### Hydatid cyst type, location and genotype

In *E. granulosus*-infected cattle, 5.21% (*n* = 19) had fertile cysts, 71.78% (*n* = 262) had infertile cysts, and in 23.01% (*n* = 84) cysts were smaller than 1 cm in diameter. Considering cyst location, 143 animals (39.72%) had cysts only in the lungs, 89 (24.72%) had cysts in the liver only and 133 (36.94%) had cysts in both organs simultaneously. A statistically significant difference was found regarding the location and type of the cysts (*χ*^2^ = 66.32, *df* = 4, *P* < 0.0001). The majority of the animals with fertile cysts had these located in the lungs only (68.42%) whereas the majority of infertile cysts were located in both organs and small cysts were mainly located in the liver (Table 2). Of the recovered hydatid cyst samples, 47.95% (*n* = 175) were genotyped. Of these, 98.86% (*n* = 173) were identified as *E. granulosus* (*s.s.*) and only 1.14% (*n* = 2) were identified as *E. ortleppi.*

**Table 2:** Frequency and percentage of organ localization according to cyst type

Cyst type	*N*	Lungs only *n* (%)	Liver only *n* (%)	Both *n* (%)
Infertile	262	107 (40.84)	40 (15.27)	115 (43.89)
Fertile	19	13 (68.42)	2 (10.53)	4 (21.05)
Small	84	23 (27.38)	47 (55.95)	14 (16.67)
Total	365	143 (39.18)	89 (24.39)	133 (36.44)

*Abbreviation*: *N/n* number of animals

### Association of hydatid cyst characteristics with animal age and co-infection with *F. hepatica*

**Fig. 2:**
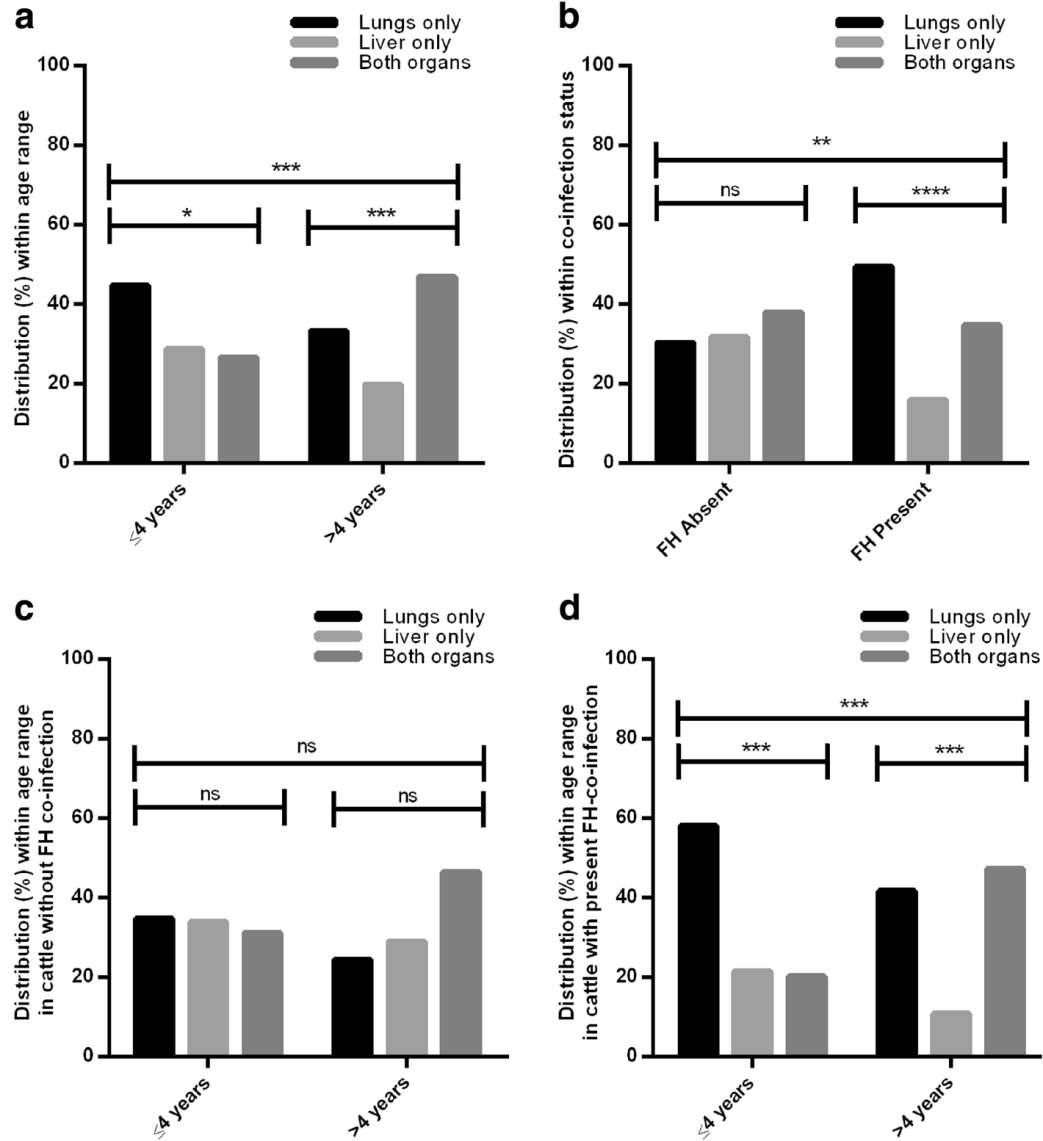
Anatomical distribution of hydatid cyst in cattle within age range (**a**); *Fasciola hepatica* (FH) co-infection status (**b**); within age range in animals without FH co-infection (**c**) and with FH co-infection (**d**). In **a** cyst anatomical distribution significantly varied among young (≤ 4 years) (*χ*^2^ = 7.973, *df* = 2, *P* = 0.0186) and old animals (> 4 years) (*χ*^2^ = 14.80, *df* = 2, *P* = 0.0006) and within age groups. In **b** a significant association was found between cyst location and FH co-infection status, where a significant difference was found in animals with FH co-infection (*χ*^2^ = 22.30, *df* = 2, *P* < 0.0001), and not in animals without FH co-infection (*χ*^2^ = 1.432, *df* = 2, *P* = 0.4883). **c** No association was found between age range and cyst location in animals absent of FH co-infection, or location within each age group. **d** In cattle with FH co-infection, a significant association was identified between age range and cyst location as well as among each age group. Data are presented as percentage (%) of the number of animals/ total examined cattle in the respective categories. *P < 0.05, **P < 0.01, ***P < 0.001, ****P < 0.0001. *Abbreviation*: ns, not statistically significant

As shown in Fig. 2a, hydatid cyst location significantly differed between age groups (*χ*^2^ = 16.30, *df* = 2, *P* = 0.0003), where the highest percentage of younger animals had cysts only in the lungs (44.68%) while the largest portion of older animals had cysts in the liver and lungs simultaneously (44.15%). Hydatid cyst in animals lacking *F. hepatica* were present in only the lungs of 30.26% of infected cattle, 31.79% had the cysts in the liver only and 37.95% in both organs. However, in the presence of concomitant *F. hepatica* infection, the proportion was significantly different (*χ*^2^ = 18.20, *df* = 2, *P* = 0.0001) with a higher percentage of animals with cysts only in the lungs (49.41%) and a lower percentage with hydatid cysts only in the liver (15.88%) and 34.71% of animals with both organs simultaneously affected (Fig. 2b). Within animals lacking *F. hepatica* co-infection, no association between location and age group (*χ*^2^ = 5.065, *df* = 2, *P* = 0.0795) or significant variation within each age group was observed (≤ 4 years: *χ*^2^ = 0.1800, *df* = 2, *P* = 0.9139; > 4 years: *χ*^2^ = 5.070, *df* = 2, *P* = 0.0793) (Fig. 2c). However, in co-infected animals, cyst location was associated with age (*χ*^2^ = 14.16, *df* = 2, *P* = 0.0008) and varied within each age group (≤ 4 years*: χ*^2^ = 15.38, *df* = 2, *P* = 0.0005; > 4 years: *χ*^2^ = 17.24, *df* = 2, *P* = 0.0002) (Fig. 2d). Specifically, in *E. granulosus* (*s.s.*)*-*infected animals, cyst location also significantly varied with *F. hepatica* co-infection (*χ*^2^ = 6.841, *df* = 2, *P* = 0.0327) but not with age (*χ*^2^ = 5.674, *df* = 2, *P* = 0.0586). Location in host varied only in animals co-infected with *F. hepatica* (*χ*^2^ = 6.209, *df* = 2, *P* = 0.0448) (Table 3).

**Table 3:** Hydatid cyst anatomical location within age range and *Fasciola hepatica* co-infection in *E. granulosus* (*s.s.*)-infected animals

FH-EG co-infection	Host age range	Lungs only *n* (%)	Liver only *n* (%)	Both organs *n* (%)	*χ* ^2^	*P*
Absent	≤ 4 years	17 (32.69)	14 (26.92)	21 (40.38)	0.6903	0.7081
	> 4 years	10 (37.04)	5 (18.52)	12 (44.44)		
	Total^a^	27 (34.18)	19 (24.05)	33 (41.77)		
Present	≤ 4 years	24 (55.81)	7 (16.28)	12 (27.91)	6.209	0.0448*
	> 4 years	22 (43.14)	3 (5.88)	26 (50.98)		
	Total^a^	46 (48.94)	10 (10.64)	38 (40.43)		
Total	≤ 4 years	41 (43.16)	21 (22.11)	33 (34.74)	5.674	0.0586
	> 4 years	32 (41.03)	8 (10.26)	38 (48.72)		
	Total	73 (42.20)	29 (16.76)	71 (41.04)		

*Statistically significant difference at *P* < 0.05

^a^Statistically significant association of cyst location and co-infection (*χ*^2^ = 6.841, *P* = 0.0327)

*Abbreviations*: *EG E. granulosus* (*s.s.*), *FH Fasciola hepatica*

No general association was found between cyst type and the age range of animals (*χ*^2^ = 1.452, *df* = 2, *P* = 0.4838) or co-infection with *F. hepatica* (*χ*^2^ = 1.64, *df* = 2, *P* = 0.4405). Conversely, the co-infection ratio (absence/presence of *F. hepatica* co-infection) of animals with infertile cysts was significantly higher in young animals (1.14, 76/54) than in older cattle (0.81, 59/73) (*χ*^2^ = 4.968, *df* = 1, *P* = 0.0258), while in the latter group there were fewer animals with small (< 1 cm) cysts in the presence of the co-infection than in the absence (24/12), resulting in a statistical association between cyst type and co-infection in this age group (*χ*^2^ = 6.349, *df* = 2, *P* = 0.0418). In animals with small hydatid cysts, *E. granulosus*-affected organs significantly varied with *F. hepatica* co-infection (*χ*^2^ = 19.45, *df* = 2, *P* < 0.0001), where the ratio in lungs, liver and both organs was 0.28 (5/18), 3.27 (36/11) and 1.8 (9/5), respectively (Table 4).

**Table 4:** Associations between hydatid cyst with or without *Fasciola hepatica* co-infection and host variables: age range and cyst location

Co-infection	Fertile				Infertile				< 1 cm diameter			
	(-/+)	Ratio	*χ* ^2^	*P*	(-/+)	Ratio	*χ* ^2^	*P*	(-/+)	Ratio	*χ* ^2^	*P*
≤ 4 years	7/3	2.33	2.554	0.11	76/54	1.41	4.968	0.0258*	26/22	1.18	13.341	0.2481
> 4 years^a^	3/6	0.5			59/73	0.81			24/12	2		
Lungs only	7/6	1.17	^b^		47/60	0.78	4.409	0.1103	5/18	0.28	19.45	<0.0001*
Liver only	2/0	–			24/16	1.63			36/11	3.27		
Both organs	1/3	0.33			64/51	1.25			9/5	1.8		

*Statistically significant difference at *P* < 0.05

^a^Statistically significant association between cysts type and co-infection in this group (*χ*^2^ = 6.349, *P* = 0.0418)

^b^*n* too small for statistical analyses

*Abbreviations*: *(-/+)* number of animals without *F. hepatica* co-infection/number of animals with *F. hepatica* co-infection

Logistic regression analysis revealed a statistically significant association between the localization of the hydatid cysts and the presence of *F. hepatica* co-infection in both, bivariate and multivariate models (Table 5). In animals with *F. hepatica* co-infection, hydatid cyst were less likely to localize in the liver than lungs, while this association remained similar in the adjusted model (OR = 0.31, *P* < 0.0001, bivariate; OR = 0.36, *P* = 0.027, adjusted model).

**Table 5:** Association between hydatid cyst location in liver and the presence of *Fasciola hepatica* co-infection

Variable	OR (95% CI)	*Z*	*P*
Co-infection^a^	0.31 (0.18–0.54)	-4.14	<0.0001
Co-infection^b^	0.36 (0.15–0.90)	-2.21	0.027

^a^Crude model (hydatid cyst location in liver *versus Fasciola hepatica* co-infection)

^b^Hydatid cyst location in liver *versus Fasciola hepatica* coinfection adjusted by age, hydatid cyst fertility and *Echinococcus granulosus* genotype

*Abbreviation*: *CI* confidence interval

## Discussion

The prevalence of *E. granulosus* and *F. hepatica* in the present study is consistent with the official national slaughter and condemnation data at abattoirs. The prevalence of *E. granulosus* infection has remained steady since 1995; however, *F. hepatica* infection has increased [27, 28]. Older animals had a higher prevalence of *E. granulosus* infection, as reported in other studies [11, 29, 30], and this prevalence in older animals remains regardless of *F. hepatica* co-infection. This could be due to an increase in time in which the animal can be exposed to *E. granulosus* [31]. For the prevalence of *F. hepatica* however, a difference was found only when *E. granulosus*-infected animals were included; no association was found between age and the proportion of animals infected. Innocent et al. [32] found an increase of liver condemnation due to *F. hepatica* in older cattle; however, the group studied had no animals over 30 months of age and disease prevalence was considerably lower [32].

The location of hydatid cysts varies among studies. In our study, most of the animals had hydatid cysts in their lungs as reported by some studies [14, 29, 33, 34]; however in others, liver was the most commonly affected organ [11, 18, 35]. A recent study in Chile, found that the proportion of organs infected with *E. granulosus* varied among geographical locations [36]. These authors have shown that of the genotyped cysts, belonging to 47.95% of *E. granulosus*-infected animals, most were identified as *E. granulosus* (*s.s.*) as has previously been reported in cattle in Chile [26]; this species is also the most common worldwide [37].

We found that hydatid cysts organ distribution varied with age, a factor not previously considered. Conversely, separating by co-infection with *F. hepatica*, a difference was found only in *F. hepatica* co-infected animals, where the proportion of animals with cysts in both liver and lungs was higher in older co-infected animals when compared with younger co-infected animals. As has been reported, older animals could have a higher number of cysts as exposure time is increased [31], which could also increase the number of organs affected.

Anatomical location was associated with co-infection status, with a decrease in liver-affected animals and an increase in lung-only-affected animals. In *E. granulosus*, portal circulation has been described as the primary route of infection by oncospheres, with a high tropism for the liver [38]. As *F. hepatica* in acute and chronic infections can damage the liver [39], it could be interfering with the establishment of *E. granulosus* in this organ. In the absence of *F. hepatica*, small hydatid cysts were mainly located in the liver, whereas when *F. hepatica* was present, small cysts were found in a larger proportion in the lungs only. Small cysts may represent either immature cysts that could develop into fertile or infertile cysts, or they could be non-viable cysts.

As reported in other studies, we found that cyst fertility significantly varied in different organs [11, 15, 29, 40], with more fertile cysts in the lungs than in other locations. No statistical association was found between cyst type and age or co-infection with *F. hepatica*, analyzed independently. However, in older cattle there was an association between cyst type and co-infection with *F. hepatica*. In the presence of the latter, there were more animals with infertile cysts and fewer with small cysts than in single infections.

Cattle cyst fertility reported in this study, is similar to that reported by other authors [11, 15], but noticeably lower than in other studies [13, 18, 33, 34, 41]. Literature data suggest that polyparasitism interactions can alter the site in host used by the parasites. For example, the antagonic relationship between *Moniliformis moniliformis* and *Hymenolepis diminuta*, in which when both parasites co-infect the gut of rats, *M. moniliformis* is able to displace *H. diminuta* to a less nutrient rich site in the small intestine; this antagonic relationship was host-specific [1]. The fact that there are different viscera affected when both *F. hepatica* and *E. granulosus* (*s.l.*) parasitize the same bovine host could be an example of an antagonic relationship, since the liver is the main organ or site in host that both parasites seek to establish themselves. The mechanisms that could explain how *F. hepatica* infection can affect the parasitized viscera by *E. granulosus* (*s.l.*) remains to be studied, since it could be *via* immune response modulation or merely the physical condition of the liver tissue after *F. hepatica* infection that makes it unsuitable for *E. granulosus* (*s.l.*) to establish. This could also be a factor that contributes to the low cyst fertility in cattle from Chile, since the liver is usually infected with *F. hepatica*. One of the limitations of our study is that we worked with natural infections, so we cannot determine the temporality of *F. hepatica* and *E. granulosus* infection in cattle, but here we have shown an effect of displacing the hydatid cysts toward the lungs and an increased proportion of small cysts associated with co-infection with *F. hepatica*.

## Conclusions

Cattle naturally co-infected with *E. granulosus* and *F. hepatica* have a lower chance of presenting hydatid cysts in the liver with an increased chance for lung cyst localization, especially for smaller hydatid cysts, suggesting that *F. hepatica* may affect the instate of *E. granulosus* in the liver. These results suggest that in cattle natural infected with *E. granulosus* and *F. hepatica*, both parasites display an antagonistic relationship.

## References

[1] Behnke JM (2008). Structure in parasite component communities in wild rodents: predictability, stability, associations and interactions .... or pure randomness?. Parasitology.

[2] Blackwell AD, Martin M, Kaplan H, Gurven M (2013). Antagonism between two intestinal parasites in humans: the importance of co-infection for infection risk and recovery dynamics. Proc Biol Sci.

[3] Moreau E, Chauvin A (2010). Immunity against helminths: interactions with the host and the intercurrent infections. J Biomed Biotechnol..

[4] Romig T, Deplazes P, Jenkins D, Giraudoux P, Massolo A, Craig PS (2017). Ecology and life cycle patterns of *Echinococcus* species. Adv Parasitol.

[5] Agudelo Higuita NI, Brunetti E, McCloskey C (2016). Cystic echinococcosis. J Clin Microbiol.

[6] Wuhrer M, Grimm C, Dennis RD, Idris MA, Geyer R (2004). The parasitic trematode *Fasciola hepatica* exhibits mammalian-type glycolipids as well as Gal(beta1-6)Gal-terminating glycolipids that account for cestode serological cross-reactivity. Glycobiology.

[7] Cucher Marcela Alejandra, Macchiaroli Natalia, Baldi Germán, Camicia Federico, Prada Laura, Maldonado Lucas, Avila Héctor Gabriel, Fox Adolfo, Gutiérrez Ariana, Negro Perla, López Raúl, Jensen Oscar, Rosenzvit Mara, Kamenetzky Laura (2015). Cystic echinococcosis in South America: systematic review of species and genotypes ofEchinococcus granulosus sensu latoin humans and natural domestic hosts. Tropical Medicine & International Health.

[8] Riesle S, Garcia MP, Hidalgo C, Galanti N, Saenz L, Paredes R (2014). Bovine IgG subclasses and fertility of *Echinococcus granulosus* hydatid cysts. Vet Parasitol.

[9] Paredes R, Godoy P, Rodriguez B, Garcia MP, Cabezon C, Cabrera G (2011). Bovine (*Bos taurus*) humoral immune response against *Echinococcus granulosus* and hydatid cyst infertility. J Cell Biochem.

[10] Paredes R, Jimenez V, Cabrera G, Iraguen D, Galanti N (2007). Apoptosis as a possible mechanism of infertility in *Echinococcus granulosus* hydatid cysts. J Cell Biochem..

[11] Addy F, Alakonya A, Wamae N, Magambo J, Mbae C, Mulinge E (2012). Prevalence and diversity of cystic echinococcosis in livestock in Maasailand, Kenya. Parasitol Res.

[12] Al Kitani FA, Al Riyami S, Al Yahyai S, Al Rawahi AH, Al Maawali M, Hussain MH (2015). Abattoir based surveillance of cystic echinococcosis (CE) in the Sultanate of Oman during 2010–2013. Vet Parasitol.

[13] Andresiuk MV, Gordo FP, Saarma M, Elissondo MC, Taraborelli A, Casalongue C (2013). *Echinococcus granulosus* genotype G1 dominated in cattle and sheep during 2003–2006 in Buenos Aires Province, an endemic area for cystic echinococcosis in Argentina. Acta Trop.

[14] Balbinotti H, Santos GB, Badaraco J, Arend AC, Graichen DÂS, Haag KL (2012). *Echinococcus ortleppi* (G5) and *Echinococcus granulosus sensu stricto* (G1) loads in cattle from southern Brazil. Vet Parasitol.

[15] Daryani A, Sharif M, Amouei A, Nasrolahei M (2009). Fertility and viability rates of hydatid cysts in slaughtered animals in the Mazandaran Province, northern Iran. Trop Anim Health Prod..

[16] Founta A, Chliounakis S, Antoniadou Sotiriadou K, Koidou M, Bampidis V (2016). Prevalence of hydatidosis and fertility of hydatid cysts in food animals in northern Greece. Vet Ital.

[17] Kamelli M, Borji H, Naghibi A (2016). Genetic identification of cattle hydatid cyst isolates from northeast and southwest of Iran. Ann Parasitol.

[18] Latif AA, Tanveer A, Maqbool A, Siddiqi N, Kyaw-Tanner M, Traub RJ (2010). Morphological and molecular characterisation of *Echinococcus granulosus* in livestock and humans in Punjab, Pakistan. Vet Parasitol.

[19] Thapa NK, Armua-Fernandez MT, Kinzang D, Gurung RB, Wangdi P, Deplazes P (2017). Detection of *Echinococcus granulosus* and *Echinococcus ortleppi* in Bhutan. Parasitol Int.

[20] Gonzalez H, Plaza J, Abalos P (1981). Fertilidad del quiste hidatídico en tres especies animales en Chile y estudio de la vitalidad de sus escólices. Bol Chil Parasitol.

[21] Poglayen G, Varcasia A, Pipia AP, Tamponi C, Parigi M, Marchesi B (2017). Retrospective study on cystic echinococcosis in cattle of Italy. Infect Dev Ctries.

[22] Scala A, Bosco A, Pipia AP, Tamponi C, Musella V, Costanzo N (2017). Cystic echinococcosis in cattle dairy farms: spatial distribution and epidemiological dynamics. Geospat Health.

[23] Abdel-Rahman EH, Abdel-Megeed KN, Abuel-Ezz NM (2003). Cross-reaction: a common trait among helminthes. J Egypt Soc Parasitol.

[24] Talaat RM, Ali NM, Elwakil HS (2013). Impact of *Schistosoma mansoni* and *Echinococcus granulosus* experimental coinfection on interleukin 10 and interferon gamma cytokines profile. Exp Parasitol.

[25] Galindo M, Schadebrodt G, Galanti N (2008). *Echinococcus granulosus*: cellular territories and morphological regions in mature protoscoleces. Exp Parasitol.

[26] Correa F, Stoore C, Horlacher P, Jimenez M, Hidalgo C, Alvarez Rojas CA (2018). First description of *Echinococcus ortleppi* and cystic echinococcosis infection status in Chile. PLoS One.

[27] Morales MA, Luengo J, Vasquez J (2000). Distribución y tendencia de la fasciolosis en ganado de abasto en Chile, 1989–1995. Parasitol Día.

[28] Luengo J, Olivares M (1995). Causales de decomiso en bovinos beneficiados en mataderos de Chile. Av Cien Vet.

[29] Negash K, Beyene D, Kumsa B (2013). Cystic echinococcosis in cattle slaughtered at Shashemanne Municipal Abattoir, south central Oromia, Ethiopia: prevalence, cyst distribution and fertility. Trans R Soc Trop Med Hyg.

[30] Rinaldi L, Maurelli MP, Veneziano V, Capuano F, Perugini AG, Cringoli S (2008). The role of cattle in the epidemiology of *Echinococcus granulosus* in an endemic area of southern Italy. Parasitol Res.

[31] Craig PS, Hegglin D, Lightowlers MW, Torgerson PR, Wang Q (2017). Echinococcosis: control and prevention. Adv Parasitol.

[32] Innocent GT, Gilbert L, Jones EO, McLeod JE, Gunn G, McKendrick IJ (2017). Combining slaughterhouse surveillance data with cattle tracing scheme and environmental data to quantify environmental risk factors for liver fluke in cattle. Front Vet Sci.

[33] Kebede W, Hagos A, Girma Z, Lobago F (2008). Echinococcosis/hydatidosis: its prevalence, economic and public health significance in Tigray region, North Ethiopia. Trop Anim Health Prod.

[34] Pednekar RP, Gatne ML, Thompson RCA, Traub RJ (2009). Molecular and morphological characterisation of *Echinococcus* from food producing animals in India. Vet Parasitol.

[35] Ibrahim MM (2010). Study of cystic echinococcosis in slaughtered animals in Al Baha region, Saudi Arabia: interaction between some biotic and abiotic factors. Acta Trop.

[36] Acosta-Jamett G, Cleaveland S, Cunningham AA (2010). Bronsvoort BMd, Craig PS. *Echinococcus granulosus* infection in humans and livestock in the Coquimbo region, north-central Chile. Vet Parasitol.

[37] Alvarez Rojas Cristian A., Romig Thomas, Lightowlers Marshall W. (2014). Echinococcus granulosus sensu lato genotypes infecting humans – review of current knowledge. International Journal for Parasitology.

[38] Brehm KK (2017). U. *Echinococcus*-host interactions at cellular and molecular levels. Adv Parasitol.

[39] Sohair IB, Eman MN (2009). Histopathological and bacteriological studies on livers affected with fascioliasis in cattle. Egypt J Comp Path Clinic Path.

[40] Mitrea IL, Ionita M, Costin II, Predoi G, Avram E, Rinaldi L (2014). Occurrence and genetic characterization of *Echinococcus granulosus* in naturally infected adult sheep and cattle in Romania. Vet Parasitol.

[41] Ernest E, Nonga HE, Kassuku AA, Kazwala RR (2008). Hydatidosis of slaughtered animals in Ngorongoro district of Arusha region, Tanzania. Trop Anim Health Prod.

